# Vitamin D Signaling in the Bovine Immune System: A Model for Understanding Human Vitamin D Requirements

**DOI:** 10.3390/nu4030181

**Published:** 2012-03-15

**Authors:** Corwin D. Nelson, Timothy A. Reinhardt, John D. Lippolis, Randy E. Sacco, Brian J. Nonnecke

**Affiliations:** 1 Department of Biochemistry, University of Wisconsin-Madison, Madison, WI 53706, USA; Email: cdnelson3@wisc.edu; 2 Ruminant Diseases and Immunology Research Unit, National Animal Disease Center, United States Department of Agriculture, Agricultural Research Service, Ames, IA 50010, USA; Email: tim.reinhardt@ars.usda.gov (T.A.R.); john.lippolis@ars.usda.gov (J.D.L.); randy.sacco@ars.usda.gov (R.E.S.)

**Keywords:** vitamin D, immunity, cattle, animal models

## Abstract

The endocrine physiology of vitamin D in cattle has been rigorously investigated and has yielded information on vitamin D requirements, endocrine function in health and disease, general metabolism, and maintenance of calcium homeostasis in cattle. These results are relevant to human vitamin D endocrinology. The current debate regarding vitamin D requirements is centered on the requirements for proper intracrine and paracrine vitamin D signaling. Studies in adult and young cattle can provide valuable insight for understanding vitamin D requirements as they relate to innate and adaptive immune responses during infectious disease. In cattle, toll-like receptor recognition activates intracrine and paracrine vitamin D signaling mechanism in the immune system that regulates innate and adaptive immune responses in the presence of adequate 25-hydroxyvitamin D. Furthermore, experiments with mastitis in dairy cattle have provided *in vivo* evidence for the intracrine vitamin D signaling mechanism in macrophages as well as vitamin D mediated suppression of infection. Epidemiological evidence indicates that circulating concentrations above 32 ng/mL of 25-hydroxyvitamin D are necessary for optimal vitamin D signaling in the immune system, but experimental evidence is lacking for that value. Experiments in cattle can provide that evidence as circulating 25-hydroxyvitamin D concentrations can be experimentally manipulated within ranges that are normal for humans and cattle. Additionally, young and adult cattle can be experimentally infected with bacteria and viruses associated with significant diseases in both cattle and humans. Utilizing the bovine model to further delineate the immunomodulatory role of vitamin D will provide potentially valuable insights into the vitamin D requirements of both humans and cattle, especially as they relate to immune response capacity and infectious disease resistance.

## 1. Introduction

The functions of vitamin D in cattle (*Bos taurus*) have been studied intensely over the last half-century [1,2,3,4,5]. Most research has been geared towards understanding the classical endocrine functions of vitamin D in regulating calcium homeostasis associated with the intense calcium demands linked to the onset of lactation. As a result the endocrine physiology of vitamin D in cattle is well described [1,2,3,4,5]. 1,25-Dihydroxyvitamin D_3_ (1,25(OH)_2_D_3_) is the biologically active form of the vitamin [4]. The well-known endocrine functions of 1,25(OH)_2_D_3_ are to stimulate calcium uptake from the intestines, bones, and kidneys [4]. Vitamin D provided in the diet or produced in the skin is rapidly transported to and sequestered by the liver where it is available for conversion to 25-hydroxyvitamin D (25(OH)D_3_). 25(OH)D_3_ moves to the blood and is carried by vitamin D binding protein and its blood concentration is the indicator of vitamin D status. Serum 25(OH)D_3_ concentrations >20 ng/mL are considered by the National Research Council (NRC) to be adequate for maintenance of calcium homeostasis [6]. Cattle naturally rely on endogenous synthesis of vitamin D_3_ in their skin for acquisition of vitamin D [7,8], however, cattle in regions such as North America or Europe often do not receive adequate sun exposure to maintain serum 25(OH)D_3_ concentrations >20 ng/mL [9]. To assure that serum 25(OH)D_3_ concentrations range from 20–50 ng/mL, the NRC recommends an intake of 20,000 international units (IUs) of vitamin D_3_ per day for lactating dairy cows [6]. Dairy producers often supplement dairy cattle with up to 40,000 IUs of vitamin D_3_ per day because of the potential benefits of additional supplementation [10]. As consequence, dairy cattle in the upper Midwest range typically have serum 25(OH)D_3_ concentrations that range from 50 to 80 ng/mL [11]. 

Results from studies investigating the role of vitamin D signaling in the bovine immune system indicate that vitamin D signaling has important implications regarding immune function and infectious disease resistance in cattle [12,13,14,15]. The immunomodulatory effects of the vitamin D pathway in cattle are relevant for human health. The Institutes of Medicine’s recommendations for vitamin D do not consider immunity-related health outcomes of vitamin D citing insufficient experimental evidence to include them in setting vitamin D requirements for people [16]. Cattle offer a unique model to increase our understanding of immunity-related health outcomes in people, and to determine the vitamin D requirements necessary for optimal immune function. Here we review the functions of the vitamin D pathway in the bovine immune system and the applicability of the bovine model for human infectious disease and vitamin D interactions. 

## 2. Vitamin D Signaling in the Bovine Immune System

1,25(OH)_2_D_3_ is a regulator of innate and adaptive immunity in cattle [13,15,17,18,19,20,21]. Targets of 1,25(OH)_2_D_3_ in the bovine innate immune system are somewhat different than in humans, but effects of 1,25(OH)_2_D_3_ on adaptive immunity are quite similar between cattle, humans, and mice (Table 1). The vitamin D-dependent immune responses in cattle are controlled through intracrine and paracrine vitamin D signaling mechanisms [13,14,15], similar to those described for human and mouse immune systems [22,23,24]. Furthermore, experiments in cattle have provided the first *in vivo* evidence for activation of an intracrine vitamin D signaling pathway in the immune system during a bacterial infection [14]. As shown in a study by Lippolis *et al*. [12], this pathway can be exploited to enhance the cow’s defense against bacterial infection, demonstrating the physiological significance of the vitamin D pathway in the immune system.

**Table 1:** Species comparison of the effects 1,25(OH)_2_D_3_ on immunity.

Immune response	Human	Mouse	Cow
**Innate Immunity**			
Cathelicidin ^a^	↑ [23,25,26]	→ [25,27]	→ [15]
CD14	↑ [28,29]	NA	→ [30]
Defensins ^b^	↑ [23,26,31]	NA	↑ [30]
iNOS	↑ [32,33]	↓ [34]	↑ [13,15]
NOD2	↑ [35]	NA	→ [30]
RANTES/CCL5	NA	NA	↑ [13,15]
**Adaptive Immunity**			
T cell proliferation	↓ [36]	↓ [37,38]	↓ [20]
IFN-γ	↓ [39,40]	↓ [41,42]	↓ [13,17,19,21]
IL-10	↑ [40,43]	↑ [44]	NA
IL-17A	↓ [39,40]	↓ [41,42,45,46]	↓ [13]
IL-17F	NA	↓ [42]	↓ [13]

^a^ Cattle have eleven cathelicidin genes compared to one cathelicidin gene in the human and mouse; ^b^ DEF4B in humans; RNA sequencing data indicates that 1,25(OH)_2_D_3_ upregulates two defensin genes in cattle. (→), Not affected by 1,25(OH)_2_D_3_; (NA), evidence for 1,25(OH)_2_D_3_ effect is not available.

### 2.1. Innate Immunity

In the innate immune system of cattle, 1,25(OH)_2_D_3_ enhances nitric oxide (NO) and RANTES/CCL5 responses of monocytes activated via toll-like receptor 4 (TLR4) [15]. Treatment of lipopolysaccharide (LPS)-stimulated monocytes with 1 and 10 nM 1,25(OH)_2_D_3_ increases inducible NO synthase (iNOS) and RANTES/CCL5 gene expression relative to LPS-stimulation alone. Upregulation of iNOS gene expression by 1,25(OH)_2_D_3_ is associated with greater NO production. Because effects of 1,25(OH)_2_D_3_ on iNOS and RANTES gene expression in resting monocytes is modest, as is LPS treatment alone, the expression of these genes seems dependent on activation of both TLR and vitamin D receptor (VDR) signaling pathways, similar to what has been described for the human innate immune response gene expression (Table 1) [31]. 

The biological relevance of 1,25(OH)_2_D_3_ induced upregulation of iNOS and RANTES in bovine monocytes has not been determined. Traditionally, NO produced by bovine macrophages is thought to be a component of their antimicrobial defense [47,48]. However, NO-dependent antimicrobial activity has not been demonstrated in bovine macrophages. Because NO is also an important signaling molecule in the immune system [49,50], 1,25(OH)_2_D_3_ induced up-regulation of NO production in activated bovine monocytes may enhance NO downstream signaling mechanisms. Increased production of RANTES, a potent chemokine [51], would presumably enhance recruitment of immune cells to sites of infection. 

Compared to the human and mouse, some effects of 1,25(OH)_2_D_3_ on innate immunity are unique to cattle. iNOS is moderately upregulated by 1,25(OH)_2_D_3_ in human peripheral blood mononuclear cells (PBMCs), and the promyelocytic HL-60 cell line [32,33]; however, anti-bacterial activity controlled by the vitamin D pathway in human macrophages is independent of NO production [23,48]. In mice, 1,25(OH)_2_D_3_ actually decreases iNOS gene expression [34]. Effects of 1,25(OH)_2_D_3_ on RANTES gene expression in mice or humans have not been reported. In humans, cathelicidin, defensin β4 (DEFB4), NOD2, and CD14 are upregulated by 1,25(OH)_2_D_3_ [25,26,35]. There are 11 cathelicidin genes and over 100 defensin genes in cattle [52]. Expression of three cathelicidin genes (CATH4, CATH5, and CATH6) with potential vitamin D-responsive elements (VDREs) are unresponsive to 1,25(OH)_2_D_3_ [15]. This is not surprising given that the functional VDRE in the cathelicidin promoter is unique to primates [27]. In addition, 1,25(OH)_2_D_3_ does not upregulate CD14 and NOD2 gene expression in bovine monocytes [30]. Therefore, effects of 1,25(OH)_2_D_3_ on bovine innate immunity differ from those in humans with regard to the regulation of cathelicidin, CD14, and NOD2 genes. Responsiveness of >100 bovine defensin genes to 1,25(OH)_2_D_3_ is currently being investigated and it appears that two bovine defensins are induced by 1,25(OH)_2_D_3_ [30]. Induction of an antimicrobial mechanism via the vitamin D pathway occurs in cattle as in humans with the exception of upregulation of cathelicidin.

### 2.2. Adaptive Immunity

Results from recent studies evaluating the effects of 1,25(OH)_2_D_3_ on adaptive immune responses in cattle, indicate that 1,25(OH)_2_D_3_ inhibits the pro-inflammatory interferon (IFN)-γ and interleukin (IL)-17 responses of antigen-specific T cells in culture [13,21] (Table 1). *Ex vivo* treatment of antigen- or mitogen-stimulated PBMC from *Mycobacterium bovis*, strain bacille Calmette Guerin (BCG)-sensitized steers with 10 nM 1,25(OH)_2_D_3_ decreases IFN-γ production approximately 3-fold [13,17,21]. Similarly, IL-17F gene expression in antigen-stimulated PBMC cultures is decreased by half when the PBMC are treated with 10 nM 1,25(OH)_2_D_3_ [13]. The IL-17A response is also inhibited, but to a lesser extent [13]. In addition, 1,25(OH)_2_D_3_ is a potent inhibitor of antigen-induced proliferation of CD4^+^ and γδTCR^+^ T cells in cattle [20]. CD4^+^ Th1 and Th17 cells secrete IFN-γ and the IL-17 cytokines, respectively [53]. γδTCR^+^ T cells, representing a significant proportion of the T cell population in cattle [54], also secrete IFN-γ and IL-17 [55,56,57]. Thus, the anti-proliferative effects of 1,25(OH)_2_D_3_ on CD4^+^ and γδTCR^+^ T cells are likely responsible for the observed decrease in IFN-γ and IL-17 responses by antigen-stimulated PBMC. 

Similar effects of 1,25(OH)_2_D_3_ have been observed with human and mouse T cells. Treatment of human and mouse CD4^+^ T cells with 1,25(OH)_2_D_3_ decreases the percentage of Th1 and Th17 cells, and consequently the production of IFN-γ and IL-17A in culture [39,40,42,45,46]. Several mouse models of Th1 and Th17-driven autoimmune disease are prevented with 1,25(OH)_2_D_3_ [41,58,59,60], and in the 1,25(OH)_2_D_3_-treated mice the Th1 and Th17 responses are diminished relative to the placebo treated mice [41,42,43,44,45,46]. The link between serum 25(OH)D_3_ and autoimmune disease risk in people is attributed to these anti-inflammatory actions of 1,25(OH)_2_D_3_ in the immune system [61,62]. How the inhibitory properties of the vitamin D pathway affect T cell-mediated immunity against pathogens *in vivo*; however, has not been determined. Because the magnitude of Th1 and Th17 responses correlates with protection against *Mycobacteria bovis* infections in cattle [63,64], attenuation of Th1 and Th17 responses by 1,25(OH)_2_D_3_ may not be beneficial in this context. However, recent work indicates that Th1-mediated immunity to *M. tuberculosis* depends on the vitamin D pathway in macrophages [65]. Further studies are needed to characterize further the effects of 1,25(OH)_2_D_3_ on adaptive immunity in cattle.

### 2.3. 1,25(OH)_2_D_3_ Synthesis in the Immune System

Research indicates that the 1,25(OH)_2_D_3_ needed to regulate innate and adaptive immune responses mentioned above is synthesized by activated immune cells. The first evidence of extra-renal 1,25(OH)_2_D_3_ synthesis was shown over two decades ago when pulmonary macrophages from sarcoidosis patients were shown to synthesize 1,25(OH)_2_D_3_ [66]. CYP27B1 gene expression in human macrophages, dendritic cells, and keratinocytes is induced by TLR recognition of pathogen-associated molecular patterns [22,23,24,67]. The enzymatic activity of CYP27B1 catalyzes the conversion of 25(OH)D_3_ to 1,25(OH)_2_D_3_. 1,25(OH)_2_D_3_ produced in human macrophages acts in an intracrine manner to induce cathelicidin and DEFB4 transcription. Similarly, bovine monocytes express CYP27B1 mRNA in response to stimulation with LPS, synthetic tri-palmitoylated lipopetide Pam3CSK4, and peptidoglycan [15]. The response of CYP27B1 mRNA in bovine monocytes is strong, increasing >40-fold in response to 100 ng/mL LPS, promoting the conversion of 25(OH)D_3_ to 1,25(OH)_2_D_3_. As a result, concentrations of 1,25(OH)_2_D_3_ synthesized by CYP27B1 are sufficient to activate monocyte VDR when 25(OH)D3_3_ is available. This was demonstrated in LPS-stimulated monocytes treated with 0–100 ng/mL 25(OH)D_3_. iNOS and RANTES responses of activated monocytes were minimal without added 25(OH)D_3_, but increased linearly with the concentration of 25(OH)D_3_ [15]. The increase in NO is also increased similarly. The effect of 25(OH)D_3_ on the monocytes was shown subsequently to depend on CYP27B1 activity, as addition of ketoconazole, a CYP27B1 inhibitor [68], blocked the effects of 25(OH)D_3_ treatment.

Induction of CYP24A1 mRNA is inhibited in LPS stimulated bovine monocytes [15]. CYP24A1 catalyzes inactivation of 1,25(OH)_2_D_3_ and its induction by 1,25(OH)_2_D_3_ normally serves to limit the concentration of 1,25(OH)_2_D_3_ [1]. LPS mediated inhibition of CYP24A1 expression in activated monocytes presumably facilitates accumulation of 1,25(OH)_2_D_3_ in activated monocytes and thus is a key regulator of 1,25(OH)_2_D_3_ action in immune cells. 

B cells are another source of 1,25(OH)_2_D_3_ in the bovine immune system. CYP27B1 gene expression is upregulated in IgM^+^ cells nearly 40-fold in antigen-stimulated PBMC cultures, relative to expression in resting (*i.e.*, nonstimulated) PBMC cultures [13]. In comparison, CYP27B1 expression in monocytes is upregulated 125-fold relative to resting PBMCs [13]. Given the higher proportion of B cells relative to monocytes in PBMC cultures, these data suggest B cells are a significant source of 1,25(OH)_2_D_3_. B cell iNOS and RANTES gene expression also increases when 25(OH)D_3_ is added to the PBMC cultures [13]; suggesting an intracrine vitamin D signaling mechanism in B cells. CYP27B1 also is upregulated in human B cells in response to B cell receptor/CD40 and TLR9 stimulation [43,69], and 1,25(OH)_2_D_3_ upregulates IL-10 in activated human B cells [43]. In contrast to activated monocytes and B cells in cattle, activated T cells do not manifest increased CYP27B1 expression [13]. Although CYP27B1 expression is not upregulated in T cells, IFN-γ and IL-17 responses of T cells were inhibited when 25(OH)D_3_ was added to cultures [13]. These results suggest that 1,25(OH)_2_D_3_ synthesis in monocytes and B cells regulates T cell responses in a paracrine manner. A similar mechanism has been proposed for control of human and mouse T cells [22,70]. 

Local control of vitamin D signaling in response to bacterial infection in cattle is evident *in vivo*. CYP27B1 gene expression is highly upregulated in mammary tissue infected with *Streptococcus* *uberis* relative to healthy tissue [14]. CYP27B1 is upregulated in the CD14^+^ cells (monocytes/macrophages) in secretions from infected mammary glands when compared to CD14^+^ cells from healthy glands or peripheral blood [14]. CD14^−^ cells from the infected gland; consisting mostly of neutrophils and a few lymphocytes [71], did not show upregulation of CYP27B1. Upregulation of CYP27B1 is associated with increased 1,25(OH)_2_D_3_ synthesis and activation of the VDR in infected mammary glands as was demonstrated by upregulation of CYP24A1 gene expression in infected mammary tissue. CYP24A1 is not upregulated in CD14^+^ cells in infected glands, supporting the observation that 1,25(OH)_2_D_3_ induced upregulation of CYP24A1 is inhibited in activated monocytes [15]. CYP24A1 is upregulated in CD14^−^ cells, even though CYP27B1 is not; suggesting that 1,25(OH)_2_D_3_ from CD14^+^ cells promotes VDR expression in CD14^−^ cells. Although much of the tissue CYP24A1 is likely in mammary epithelial cells, the contribution of these cells to CYP27B1 and CYP24A1 expression in tissue was not evaluated. Mammary epithelial cells may be another source of CYP27B1 in the mammary gland given they express CYP27B1 and synthesize 1,25(OH)_2_D_3_ [72,73]. Both iNOS and RANTES are up-regulated in tissue and CD14^+^ cells from infected glands [14], but whether their expression is dependent solely on 1,25(OH)_2_D_3_ synthesis in infected glands is uncertain. Regardless, increased expression of CYP27B1 and CYP24A1 in infected mammary glands provides strong *in vivo* evidence for local control of vitamin D signaling during the innate response to bacterial infection.

The physiological significance of vitamin D signaling in host defense has been demonstrated recently in cattle [12]. It was hypothesized that intra-mammary infusion of 25(OH)D_3_ would be an effective treatment for mastitis in dairy cattle because the vitamin D signaling pathway is present in mammary glands during mastitis [14] and 25(OH)D_3_ concentrations in mammary secretions are low (<1 ng/mL) relative to serum 25(OH)D_3_ concentrations (>50 ng/mL) [74,75]. To test this hypothesis, dairy cattle with experimentally-induced, bacterial mastitis were infused intra-mammary with 25(OH)D_3_ (100 μg in fetal bovine serum) or a placebo (fetal bovine serum) daily [12]. Cows receiving 25(OH)D_3_ had significantly lower bacterial counts, reduced numbers of leukocytes in mammary secretions from infected glands, and lower body temperatures than control cows demonstrating that 25(OH)D_3_ can limit the severity of bacterial-induced mastitis. No changes were observed in serum 25(OH)D or 1,25(OH)_2_D, suggesting that endocrine system did not have a role in the effects of 25(OH)D_3_ on the infection. 25(OH)D_3_ may have acted directly through the VDR in the infected mammary gland; however, the observed induction of CYP27B1 gene expression in the infected mammary gland suggests that 25(OH)D_3_ was likely converted to 1,25(OH)_2_D_3_ to elicit the observed effects. Although treatment with 25(OH)D_3_ did not eliminate the infection, a significant beneficial effect of the vitamin D signaling pathway on the innate immune system was demonstrated.

### 2.4.Vitamin D Requirements of the Immune System

The vitamin D signaling pathway is clearly present in the immune system in humans and cattle alike [13,14,15,22,23], and as shown in cattle, that pathway has clear physiological implications in host defense [12]. So what serum 25(OH)D_3_ concentration range promotes optimal immune function, or for that matter, overall health in general? Is immune function suppressed by unnaturally high serum 25(OH)D_3_ concentrations? As discussed later in this review, data suggests that sustained high serum 25(OH)D_3_ concentrations (>100 ng/mL) during the neonatal period may alter gene and protein expression associated with development of adaptive immune responses in calves vaccinated at an early age [76] and their subsequent response to experimental challenge with bovine respiratory syncytial virus [77]. 

Questions regarding the range in 25(OH)D_3_ concentrations that represents optimal vitamin D status persist with considerable debate regarding vitamin D requirements for both humans [78] as well as young and adult cattle. The best indication of what serum 25(OH)D_3_ concentrations promote optimal health are likely represented by levels achieved in a natural setting that provides abundant exposure of the skin to sunlight. For cattle and humans, these concentrations likely lie somewhere between 30 and 100 ng/mL [79,80]. Epidemiological evidence from the human population suggests this may be the case as the prevalence of upper respiratory tract infection, influenza A, tuberculosis and several autoimmune diseases are inversely correlated with vitamin D status [81,82,83,84,85,86]. These findings support the contention that serum 25(OH)D_3_ concentrations >30 ng/mL are needed to support optimal immune function in humans [62,78,87,88]. Given the similarities between humans and cattle regarding vitamin D physiology, the same is likely true for cattle. 

In the endocrine system, the rate that 1,25(OH)_2_D_3_ appears in the circulation is dependent on CYP27B1 and CYP24A1 expression in the kidneys, and 25(OH)D_3_ and 1,25(OH)_2_D_3_ concentrations in the circulation [1]. CYP27B1 and CYP24A1 are tightly regulated in the kidneys by endocrine factors (e.g., PTH, 1,25(OH)_2_D_3_) to assure 1,25(OH)_2_D_3_ concentrations necessary for Ca and P homeostasis are maintained [89]. In the intracrine vitamin D signaling mechanism of the macrophage, the rate of appearance of 1,25(OH)_2_D_3_ appears to be associated with the expression of CYP27B1 and CYP24A1 and 25(OH)D_3_ and 1,25(OH)_2_D_3_ concentrations in the macrophage. Furthermore, CYP27B1 and CYP24A1 are regulated by immune stimuli (e.g., TLR ligands, IFN-γ) [15,23,65,67] and unlike the endocrine system, their expression is not adjusted further to achieve 1,25(OH)_2_D_3_ concentrations necessary for optimal macrophage function. Therefore, diffusion of 1,25(OH)_2_D_3_ from the cell over time becomes critical and necessary for an optimal response. For this reason, serum 25(OH)D_3_ concentrations of 20–30 ng/mL that support the classic endocrine function of vitamin D may be too low to support optimal immune function yet sufficient for bone and calcium homeostasis. Consequently, when recommending vitamin D requirements for optimal health in humans and cattle it is essential that the intracrine vitamin D signaling mechanisms be considered as well as the endocrine effects.

Because approaches promoting optimal immune function in cattle reduce reliance on antimicrobials, improve the health and productivity of cattle, and as a consequence food safety, the potentially beneficial immunomodulatory effects of vitamin D in bovine warrant continued investigation. Furthermore, cattle provide a valuable model for estimating the vitamin D requirements of the human immune system. A concern of the mouse model is that the vitamin D requirements of the mouse differ from those of humans and cattle. This difference is likely because mice are nocturnal and do not rely on endogenous synthesis of vitamin D_3_ in the skin to assure vitamin D adequacy. In contrast, vitamin D physiology and the functions of the vitamin D pathway in modulating the immune response capacity of humans and cattle are quite similar, with the exception of some genes regulated by the vitamin D pathway in macrophages. Recognizing these differences, cattle still offer an advantage in that they can be experimentally infected with viral and bacterial pathogens and their serum 25(OH)D_3_ concentrations (*i.e.*, vitamin D status) can be experimentally manipulated to correspond to ranges considered relevant to human health. As demonstrated recently, the ability to experimentally infect cattle has proven to be a valuable approach for increasing our understanding of the physiological significance of the vitamin D pathway in host defense [54]. Also, serum 25(OH)D_3_ concentration, reflective of vitamin D status, can be experimentally controlled to mimic vitamin status ranging from deficiency to the point of toxicity [74,90,91,92]. For obvious ethical reasons, manipulation of vitamin D status to represent deficiency or toxicity is not possible in humans. The ability to investigate the entire range in the bovine model provides an opportunity to determine the range of serum 25(OH)D_3_ concentrations promoting optimal immune function. 

### 2.5. Preruminant Calf as a Model for Investigating the Effects of Vitamin D in the Neonate

When considering the applicability of the bovine model for the study of the effects of vitamin D, the preruminant calf offers a particularly valuable approach for examining the effects of extremes in vitamin D status on the growth, immune function, and health of the neonate, including the human infant. Unlike studies in human infant, those in the neonatal calf offer several benefits including the establishment of extremes in vitamin D status, the opportunity to examine for an extended period the effects of vitamin D status on, not only mineral metabolism and growth performance, but also immune responses elicited early vaccination and disease severity and duration associated with experimentally-induced viral and/or bacterial infections. 

Serum 25(OH)D_3_ concentrations in the newborn calf can be manipulated in a predictable fashion by subcutaneous administration of 25(OH)D_3_ [92] or by altering the amount of vitamin D in the diet. Using the former approach, it was possible to maintain for several weeks calves with low vitamin D status (mean serum 25(OH)D_3_ <30 ng/mL) or high-normal status (mean serum 25(OH)D_3_ >60 ng/mL). These results were the first to demonstrate that it is possible to control predictably vitamin D status during the neonatal period and to suggest that the young calf may be a useful model for studying effects of vitamin D on growth, development, and immune function in the neonate. Results from a more recent study [76] suggest that vitamin D status of preruminant calves can also be manipulated by feeding custom-formulated milk replacers supplemented with varying amounts of vitamin D_3_. Because calves in these studies received colostrum at birth and were subsequently fed milk replacers with defined composition, it was possible to establish groups of calves representing deficiency (25(OH)D_3_ <25 ng /mL of serum), normal status (25–100 ng/mL of serum) and high vitamin D status (>100 ng/mL) status. All calves were vaccinated with BCG at approximately 7 day of age when differences in vitamin D status were established. Examination of *ex vivo* antigen-induced recall responses of PBMC collected at 56 day of age indicated that vitamin D status influences antigen (*i.e.*, *M. bovis* derived purified protein derivative) induced IL-4, RANTES, IL-17A, and IL-17F gene expression as well as the number of IFN-γ secreting cells. Interestingly, these responses by low and high status calves were comparable and differed from responses of calves with serum 25(OH)D_3_ concentrations within the normal range. Although preliminary, results suggest that the vitamin D status during the neonatal period influences aspects of the adaptive immune response associated with the development of protective immunity induced by early vaccination. New studies are necessary to determine if these vitamin D status-dependent effects are unique to the BCG sensitization/PPDb recall system. Additionally, the calf model provides the opportunity to evaluate the effects of vitamin D status on disease development elicited by experimental challenge with virulent strains of *M. bovis*. In young calves, lung pathology elicited by experimental infection with *M. bovis* resembles that of the human more so than that of the mouse [93,94]. 

As noted above, the calf model has considerable potential for studies evaluating the effects of vitamin D status on disease development in the neonate. Experimental RSV infection in calves resembles RSV infection in infants, having similar microscopic lung lesion, including a prominent neutrophils infiltrate, and comparable immune pathology [95,96]. Recent research [77] evaluated the effects of vitamin D status on the response of preruminant calf to experimental infection with RSV. During the first 10 wk postpartum, calves were fed milk replacers with differing amounts of vitamin D_3_ resulting in two treatment groups representing low (25(OH)D_3_ <25 ng/mL) and high vitamin D status (25(OH)D_3_ >100 ng/mL). At 70 day of age, calves in both groups were aerosol-challenged with RSV and necropsied 7 days later. Disease development, based on gross and microscopic lesions in the lung, was similar in both groups; however, it was observed that cytokines often found to be suppressed by the vitamin D pathway *in vitro* are either increased significantly (IL-12p40) or unaffected (IFN-γ) in the lungs of RSV infected, high vitamin D status calves. Furthermore, expression of the pro-inflammatory cytokine, IL-8 (CXCL8) was enhanced (*P* = 0.10) in calves with high plasma 25(OH)D_3_ concentrations. These results suggest that vitamin D status modulates inflammatory cytokine gene expression in the virus-infected lung of the calf. Failure to observe effects of vitamin D status on lung pathology may be because the study was terminated at the peak of disease response. Future studies will address the effects of vitamin D status on duration and recovery following experimental infection of the lung. 

Infection models in newborn calves offer an opportunity to study the effects of vitamin D status on host responses to bacterial and viral pathogens that are relevant to human health. New studies evaluating the effects of vitamin D status on immune function and disease severity/duration in the pre-ruminant calf will provide important information necessary for establishing the vitamin D requirements of the immune system of the newborn.

## 3. Conclusion

Cattle offer a valuable model for understanding vitamin D requirements for optimal health in humans. Cattle, like humans, rely on endogenous synthesis of vitamin D_3_ in their skin for acquisition of vitamin D, and the endocrine physiology of vitamin D is quite similar between cattle and humans. In addition, many naturally occurring pathogens in cattle are similar to those affecting humans (e.g., TB, RSV, mycoplasma) and thus, the bovine model is useful for investigating the effects vitamin D on the pathogenesis of infectious diseases. Recent observations indicate that the vitamin D pathway influences innate and adaptive immune responses in cattle as it does in humans. Research examining the effects of vitamin D on the severity of experimentally induced mastitis in dairy cattle has provided *in vivo* evidence for an intracrine mechanism of vitamin D signaling pathway in macrophages, and has demonstrated the potential for vitamin D to reduce the severity of bacterial infection of the bovine mammary gland. Research also suggests that experimentally induced alterations in the vitamin D status of the preruminant calf influence cytokine gene and protein expression associated with the adaptive (*i.e.*, antigen-specific) immune response as well as its response to an aerosol challenge with BRSV. 

Given the fundamental differences between intracrine vitamin D signaling within the immune system and the classical endocrine role of vitamin D, it is clear that the vitamin D requirements of the bovine and human immune systems warrant further investigation. The bovine model offers an experimental approach for investigating the influence of vitamin D on immune system and infectious disease response, thus providing insight into the role of vitamin D in promoting health in humans as well as cattle.
